# Development of a cell system for siRNA screening of pathogen responses in human and mouse macrophages

**DOI:** 10.1038/srep09559

**Published:** 2015-04-01

**Authors:** Ning Li, Jing Sun, Zachary L. Benet, Ze Wang, Souhaila Al-Khodor, Sinu P. John, Bin Lin, Myong-Hee Sung, Iain D. C. Fraser

**Affiliations:** 1 Signaling Systems Unit, Laboratory of Systems Biology, National Institute of Allergy and Infectious Diseases, National Institutes of Health, Bethesda, MD 20892, USA; 2 Lymphocyte Biology Section, Laboratory of Systems Biology, National Institute of Allergy and Infectious Diseases, National Institutes of Health, Bethesda, MD 20892, USA; 3 Laboratory of Receptor Biology and Gene Expression, National Cancer Institute, National Institutes of Health, Bethesda, MD 20892, USA

## Abstract

Macrophages play a critical role in the innate immune response to pathogen infection, but few tools exist for systematic dissection of these responses using modern genome-wide perturbation methods. To develop an assay platform for high-throughput analysis of macrophage activation by pathogenic stimuli, we generated reporter systems in human and mouse macrophages with dynamic readouts for NF-κB and/or TNF-α responses. These reporter cells show responsiveness to a broad range of TLR ligands and to gram-negative bacterial infection. There are significant challenges to the use of RNAi in innate immune cells, including efficient small RNA delivery and non-specific immune responses to dsRNA. To permit the interrogation of the macrophage pathogen response pathways with RNAi, we employed the stably expressed reporter genes to develop efficient siRNA delivery protocols for maximal target gene silencing with minimal activation of the innate macrophage response to nucleic acids. We demonstrate the utility of these macrophage cell systems for siRNA screening of pathogen responses by targeting components of the human and mouse TLR pathways, and observe species-specific perturbation of signaling and cytokine responses. Our approach to reporter cell development and siRNA delivery optimization provides an experimental paradigm with significant potential for developing genetic screening platforms in mammalian cells.

Macrophages are central to the innate immune response to bacterial, parasitic and viral pathogens and they respond to these infectious stimuli through a range of pattern recognition receptors (PRRs) that interact with conserved motifs, such as invariant structural components of bacterial cell walls (e.g., lipopolysaccharide (LPS) or peptidoglycan) or pathogen-specific nucleic acid motifs^1^,^2^,^3^. Classes of PRRs include the membrane-associated toll-like receptors (TLRs), the cytosolic Nod-like receptors (NLRs), and the RIG-I-like receptors (RLRs)^4^. Engagement of these host receptors leads to the activation of components in one or more of the nuclear factor kappa B (NF-κB), interferon regulatory factor (IRF), and mitogen activated protein kinase (MAPK) dependent transcription factors families, leading to the subsequent expression of numerous inflammatory cytokines and immune mediators such as tumor necrosis factor-α (TNF-α) and type I interferons^5^.

The discovery of RNA interference (RNAi) and the major advances in the understanding of small RNA biology in the past decade have provided researchers with an invaluable tool for wide-scale and rapid genetic screening^6^,^7^. RNAi takes advantage of the endogenous microRNA processing machinery to silence mRNA transcripts by introduction of a short interfering (si)RNA complementary to the target gene mRNA, permitting the systematic evaluation of gene product dependencies in a given biological system through targeted inhibition of gene expression^8^. However, there are significant challenges to the use of this technology in innate immune cells, including efficient small RNA delivery and non-specific immune responses to dsRNA^9^,^10^,^11^,^12^,^13^. These challenges have led to very few reports of siRNA-based screens in innate immune cells as opposed to more easily employed fibroblast or mesenchymal cell lines that do not have the same crucial roles in host defense as the macrophage.

In this study, we report the development of a highly optimized cell-based platform for siRNA screening in the most commonly used human and mouse macrophage model cell lines, THP1^14^ and RAW264.7^15^. The engineered macrophages provide readouts for NF-κB and/or TNF-α activation. We show that the stably integrated reporters respond to a broad range of TLR ligands and to infection with the gram-negative bacterium, *Burkholderia cenocepacia*. Using the stably expressed reporters as target genes to screen for optimal siRNA delivery conditions, we identify protocols in both human and mouse macrophage cells that achieve consistently high target gene knockdown with no detectable non-specific dsRNA response. To validate this platform, we targeted canonical components of the human and mouse TLR pathways with numerous independent siRNAs per gene, and demonstrate perturbation of signaling and inflammatory cytokine responses to specific TLR ligands.

## Results

### Development of a dual-promoter lentiviral expression system to generate macrophage reporter cell lines for RNAi screening

**Figure 1:**
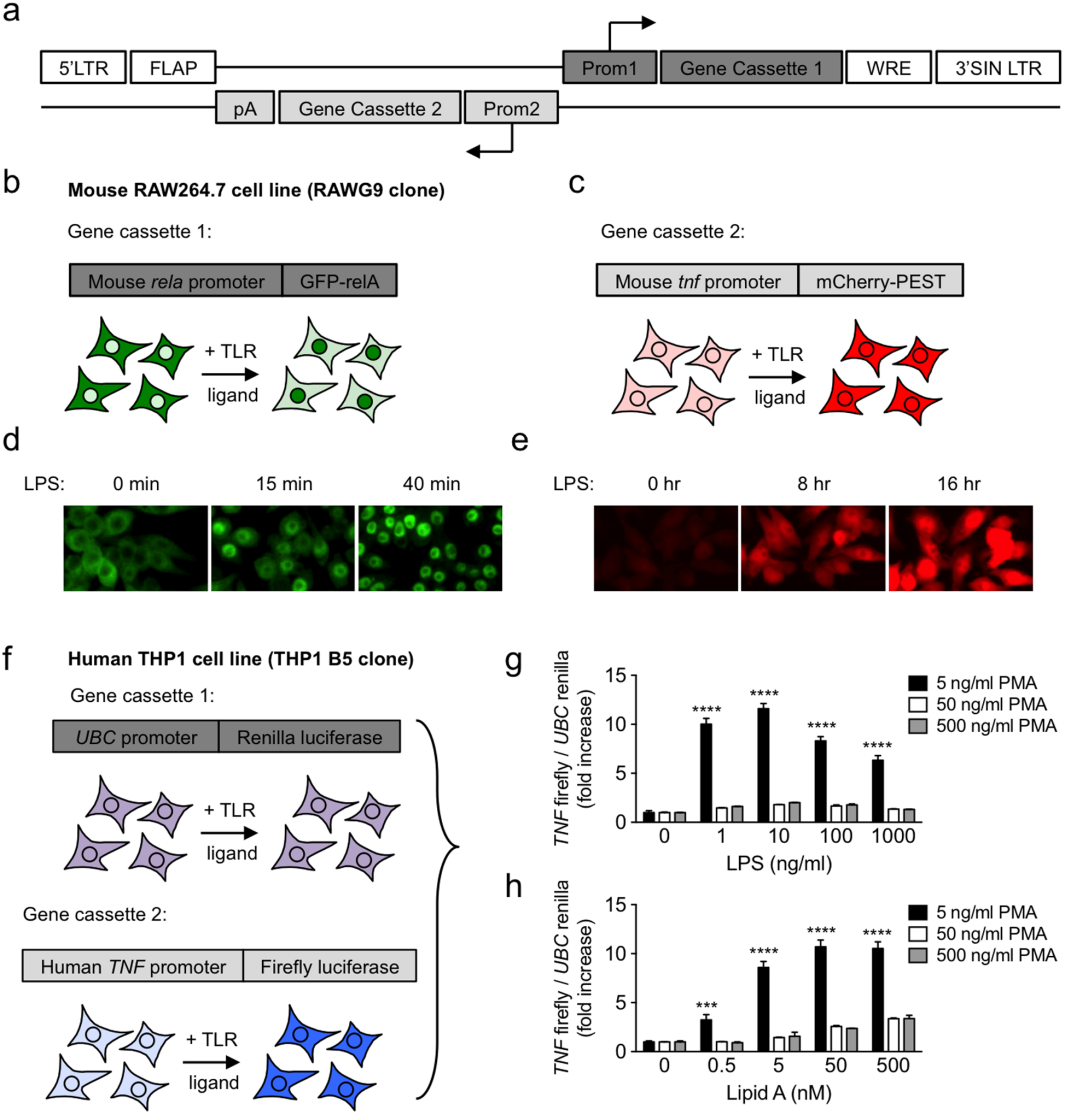
Generation of mouse and human macrophage TLR pathway reporter cell lines for siRNA screening applications. (a) Design of a dual-promoter lentiviral vector for expression of TLR pathway reporters. (b + c) Gene cassettes in the mouse RAW G9 reporter clone containing (b) the mouse *rela* promoter driving expression of a GFP-relA fusion protein and (c) the mouse *tnf* promoter driving expression of an mCherry-PEST fusion protein. (d) Cytosol-to-nuclear translocation of the GFP-relA fusion in RAW G9 cells up to 40 min after treatment with 10 ng/ml LPS (e) Increased *tnf* promoter-driven mCherry expression in RAW G9 cells up to 16 hr after treatment with 10 ng/ml LPS (f) Gene cassettes in the human THP1 B5 reporter clone containing the human *UBC* promoter driving constitutive expression of renilla luciferase and the human *TNF* promoter driving TLR ligand-inducible expression of firefly luciferase. (g–h) Human TNF-α reporter responses in THP1 B5 cells differentiated with different doses of PMA for 72 hr, and stimulated for 4 hr with a range of (g) LPS or (h) Lipid A doses. Data are representative of three experiments (g, h; mean + s.d.). ***P < 0.001, ****P < 0.0001 (two-tailed t test).

To allow the systematic functional testing of the components of PRR signaling pathways in a relevant innate immune cell type, we first developed a dual promoter lentiviral expression vector to establish human and mouse macrophage cell lines expressing reporters responsive to PRR activation (Fig. 1a). Our aim was to develop dynamic readouts for the most typically activated signaling pathway (NF-κB) and/or inflammatory cytokine (TNF-α) responses to pathogenic stimuli, and to include means to correct for cell number variation, which is particularly important for high-throughput screening applications^7^,^16^. Using this lentiviral platform, we created a RAW264.7 mouse macrophage cell line expressing two fluorescent biosensors for ‘high content’ image-based screening (RAW G9 clone; Fig. 1b–c). The first gene cassette expresses GFP fused to the relA NF-κB transcription factor driven by its endogenous promoter. This fusion protein partitions to the cytoplasm in unstimulated cells, and translocates to the nucleus in the first 40 min after activation with LPS (Fig. 1d, Supplementary Videos 1 + 3). The second gene cassette contains the murine TNF-α promoter driving expression of the red fluorescent protein mCherry, fused to a destabilizing PEST sequence that reduces the protein's half-life in the cell to provide a dynamic readout of TNF-α promoter activity. This biosensor shows a significant increase in mCherry expression in response to LPS activation of the engineered RAW G9 cells (Fig. 1e, Supplementary Videos 2 + 3). We used a similar strategy to create a THP1 human monocyte cell clone (THP1 B5) expressing firefly luciferase driven by the human TNF-α promoter combined with renilla luciferase driven by a ubiquitin promoter to provide a normalization factor for cell number variability in assay wells (Fig. 1f). Thus, the firefly/renilla ratio in this cell line after TLR stimulation provides a specific measure of the human TNF-α promoter activity. THP1 cells normally grow in suspension, but they can be differentiated into an adherent and more macrophage-like state by treatment with PMA^14^,^17^. This also induces increased expression of several key macrophage functional genes such as CD14 and macrophage scavenger receptors (data not shown). We found that the concentration of PMA and length of incubation time led to considerable differences in the fold-increase in signal from the human TNF-α promoter reporter in the THP1 B5 cell line. Activation of the human TNF-α promoter driven firefly luciferase expression over a range of LPS doses was substantially higher after 72 hr of differentiation with a low concentration of 5 ng/ml PMA, compared to either 50 or 500 ng/ml PMA concentrations (Fig. 1g). A similar pattern of increased responsiveness at low PMA levels was observed using the purified LPS component Lipid A (Fig. 1h), and is consistent with previous reports of higher sensitivity in THP1 cells differentiated with low PMA concentration^18^. Moreover, comparison of the length of differentiation time showed the signal increase in response to TLR ligand activation was more substantial after 48–72 hr of differentiation than after 24 hr, presumably due to the time required for the cells to adopt a more macrophage-like state (data not shown). Thus, a 72 hr incubation with 5 ng/ml PMA was selected as the optimal differentiation condition for the THP1 B5 cell line.

### Mouse and human macrophage reporter cell lines respond to a broad range of TLR ligands

**Figure 2:**
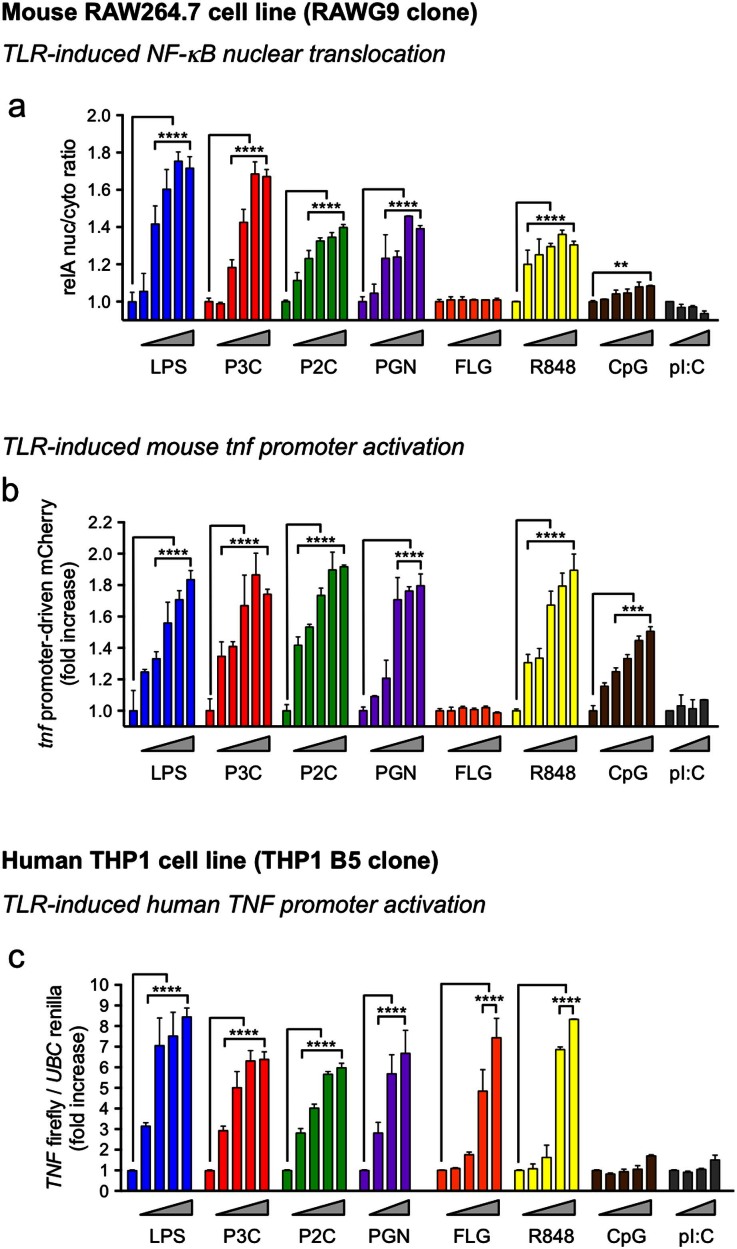
TLR ligand dose responses in mouse and human macrophage reporter cell lines. (a + b) Dose response of (a) cytosol-to-nuclear translocation of the GFP-relA fusion at 30 min (120 min for CpG) and (b) *tnf* promoter-driven mCherry expression at 12 hr after treatment of RAW G9 cells with LPS (0, 0.5, 1, 5, 12.5 and 25 ng/ml), P3C (0, 62.5, 125, 250, 500 and 1000 nM), P2C (0, 7.8, 31.25, 62.5, 125 and 250 nM), PGN (1, 15, 37.5, 75, 150 and 300 ng/ml), FLG (0, 0.1, 1, 10, 100 and 1000 ng/ml), R848 (0, 0.37, 0.75, 1.5, 3 and 6 μM), CpG (0, 0.35, 0.7, 1.4, 2.8 and 5.6 μg/ml) or pI:C (0, 12.5, 25 and 50 μg/ml). (c) Dose response of the *TNF* firefly/*UBC* renilla luciferase expression ratio in THP1 B5 cells at 4 hr after treatment with LPS (0, 0.1, 1, 10 and 100 ng/ml), P3C (0, 1, 10, 100 and 1000 nM), P2C (0, 0.1, 1, 10 and 100 nM), PGN (0, 1, 10 and 100 μg/ml), FLG (0, 1, 10, 100 and 1000 ng/ml), R848 (0, 0.1, 1, 10 and 50 μg/ml), CpG (0, 1, 10, 100 and 1000 nM) or pI:C (0, 1, 10 and 100 μg/ml). Data are representative of three experiments (a–c; mean + s.d.). **P < 0.01, ***P < 0.001, ****P < 0.0001 (two-tailed t test).

To assess the responsiveness of the RAW G9 and THP1 B5 macrophage cell lines, we stimulated them with a range of stimuli for different TLRs: Lipopolysaccharide (LPS; TLR4), Pam3CSK4 (P3C; TLR2/1), Pam2CSK4 (P2C; TLR2/6), peptidoglycan (PGN; TLR2/6), flagellin (FLG; TLR5), resiquimod 848 (R848; TLR7/8), CpG DNA (CpG; TLR9) and poly I:C (pI:C; TLR3). We chose a range of 5–6 concentrations for each ligand to assess dose-responsiveness (see Fig. 2 legend). The mouse macrophage RAW G9 cells showed NF-κB and TNF-α reporter responses to all the tested TLR ligands except flagellin and poly I:C (Fig. 2a + b). This is consistent with microarray analysis of these cells that finds no significant expression of either TLR5 or TLR3, but detectable expression of the receptors for the other tested TLR ligands (Supplementary Fig. 1). The human macrophage THP1 B5 cells show a TNF-α reporter response to all the tested TLR ligands except CpG and pI:C (Fig. 2c), which is again consistent with undetectable transcript levels for their respective receptors, TLR9 and TLR3 in both parental THP1 cells and the THP1 B5 reporter clone (Supplementary Fig. 1).

**Figure 3:**
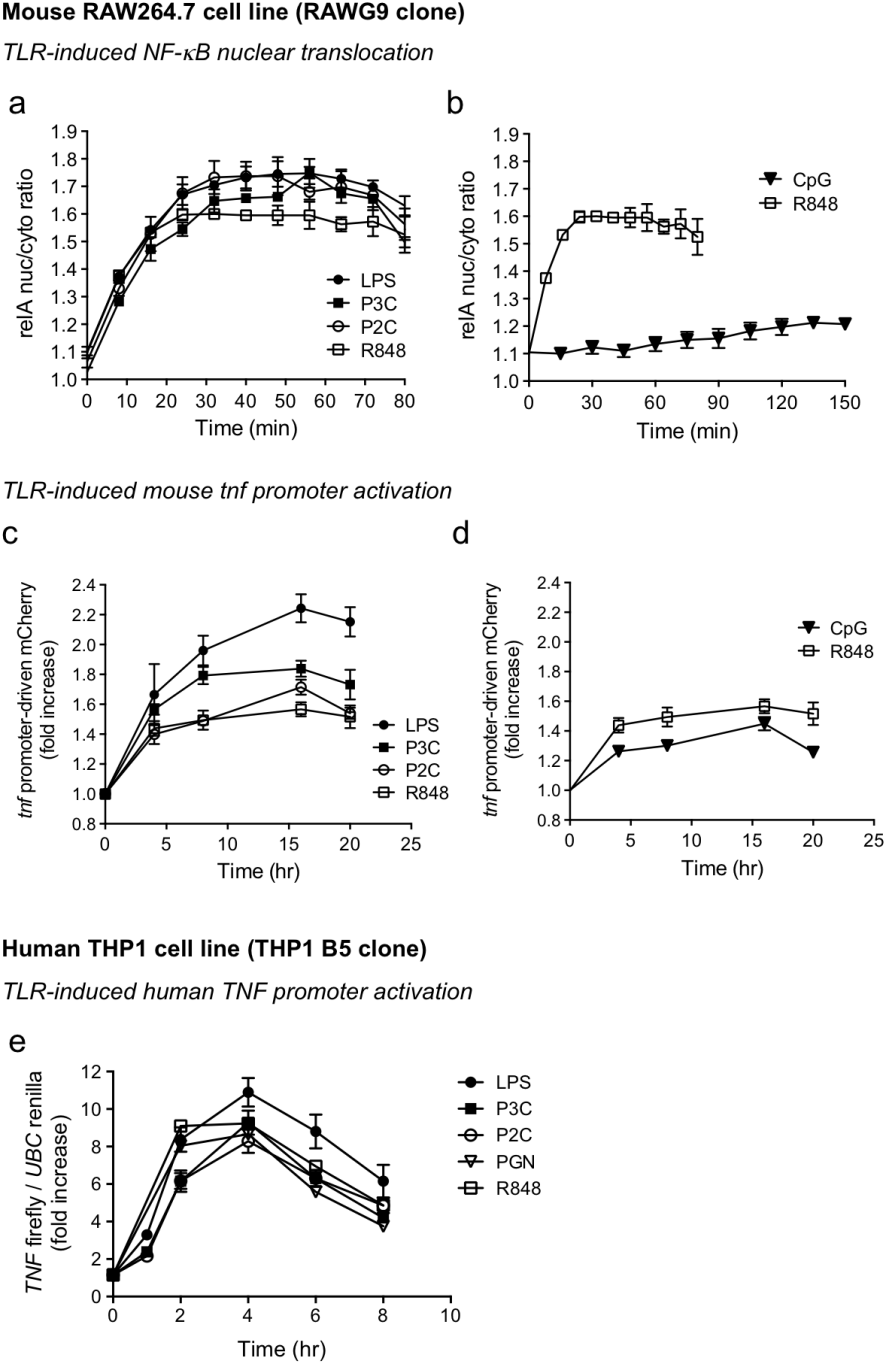
TLR ligand response kinetics of mouse and human macrophage reporter cell lines. (a–d) Time course of (a + b) cytosol-to-nuclear translocation of the GFP-relA fusion and (c + d) *Tnf* promoter-driven mCherry expression in RAW G9 cells after treatment with (a + c) 10 ng/ml LPS, 250 nM P3C, 125 nM P2C and 3 μM R848 and (b + d) 100 nM CpG and 3 μM R848. (e) Time course of the *TNF* firefly/*UBC* renilla luciferase expression ratio in THP1 B5 cells after treatment with 10 ng/ml LPS, 100 nM P3C, 10 nM P2C, 10 μg/ml PGN and 10 μg/ml R848. Data are representative of three experiments (a–e; mean + s.d.)

Based on the dose-response data for the TLR ligands that gave responses in the RAW G9 and THP1 B5 reporter cells, we selected single ligand concentrations and assessed time-courses of activation for each reporter readout. In the mouse RAW G9 cells, the NF-κB response to LPS, P3C, P2C and R848 showed similar kinetics with a peak in the nuclear/cytosolic relA ratio at around 40 min (Fig. 3a). In contrast, the response to CpG showed slower kinetics with a peak closer to 2 hr (Fig. 3b), possibly due to the time required for this ligand to fully activate the endosomal TLR9 receptor. However, it is noteworthy that the receptor for R848 (TLR7 or TLR8) is also endosomal, but is activated with faster kinetics (Fig. 3b). The TNF-α reporter response in the RAW G9 cells peaked around 16 hr for LPS, P3C, P2C and R848 (Fig. 3c). Although the initial rate of activation of the TNF-α reporter by CpG was slower, consistent with the slower activation of NF-κB (Fig. 3b), the peak of TNF-α reporter activation was still reached at 16 hr, albeit to a lower maximum level than the other TLR ligands (Fig. 3d). We have previously shown that the PEST sequence, which confers a half-life of approximately 1 hr on the mCherry reporter protein, allows for a close correlation between mCherry intensity and synthesis rate, such that the measured fluorescence provides a dynamic reflection of TNF-α promoter activity^19^. This prior study also demonstrated that upon TLR activation of the RAW G9 clone, the nuclear/cytosolic relA ratio diminished close to basal levels by 5 hr after activation, while the TNF-α reporter signal was reduced after 24 hr^19^. In the human THP1 B5 cells, we observed a faster activation of the TNF-α luciferase reporter, with a signal peak at 4 hr (Fig. 3e). This could reflect faster activation kinetics of the TNF-α promoter in human cells and/or more sensitive detection of the firefly luciferase readout compared to mCherry fluorescence.

### Mouse and human macrophage reporter cell lines respond to gram-negative bacterial infection

**Figure 4:**
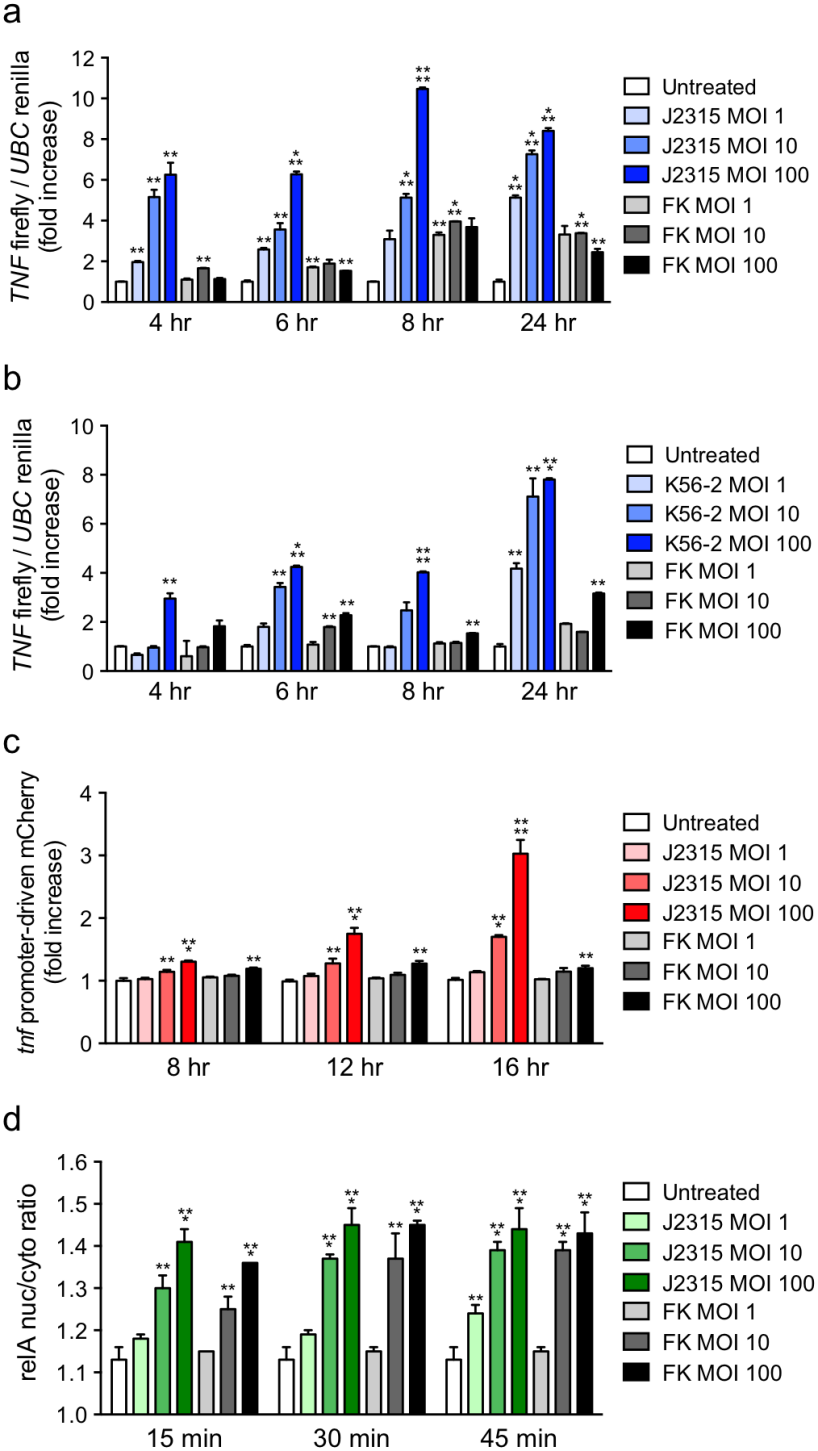
Responses to gram-negative bacterial infection in mouse and human macrophage reporter cell lines. (a + b) Dose response and time course of the *tnf* firefly/*ubc* renilla luciferase expression ratio in THP1 B5 cells infected with either the (a) J2315 or (b) K56-2 strain of *B. cenocepacia*. (c + d) Dose response and time course of the (c) *tnf* promoter-driven mCherry expression and (d) cytosol-to-nuclear translocation of the GFP-relA fusion in RAW G9 cells infected with the J2315 strain of *B. cenocepacia*. MOI: Multiplicity of infection (bacteria per cell). FK: formalin-killed bacteria. Data are representative of two experiments (a–d; mean + s.d.). **P < 0.01, ***P < 0.001, ****P < 0.0001 (two-tailed t test).

To assess whether the reporters in the described human and mouse macrophage cells were responsive to pathogen infection, we challenged the cells with the gram-negative bacterium *Burkholderia cenocepacia*. Strains of the *B. cenocepacia* complex are opportunistic pathogens that can cause serious infections in immune compromised patients, particularly those suffering from cystic fibrosis (CF) or chronic granulomatous disease (CGD)^20^,^21^,^22^,^23^,^24^. We infected the human THP1 B5 cells with increasing MOIs of two different bacterial strains that have been isolated from infected patients, J2315 and K56-2. In both cases, we observed a bacterial MOI-dependent increase in the activation of the human TNF-α reporter (Fig. 4a + b). In a 24 hr time course, the peak reporter activation was usually observed at 24 hr for both strains, except at a high MOI of 100 for the J2315 strain, which showed peak activation at 8 hr. In previous work, we have shown that at an MOI of 1, the J2315 strain escapes the host endosome and begins to replicate in the cytosol by 8 hr, reaching a peak of replication at 20–24 hr^25^. This is consistent with the reporter activation kinetics observed at low and moderate MOIs of 1 and 10. We also observed up to 4-fold activation of the human TNF-α reporter with a high MOI of formalin killed (FK) bacteria. Although killed bacteria cannot replicate in the host cell, the activation of the TNF-α reporter at early time points by FK bacteria is consistent with presence of TLR ligands on the killed bacterium surface, while the activation at later times could correlate with the detection of bacterial nucleic acid PAMPs upon bacterial degradation, as we have previously observed significant colocalization of FK bacteria with lysosomal markers at these time points^25^.

Infection of the mouse RAW G9 cells with *B. cenocepacia* also led to activation of the stably expressed NF-κB and TNF-α reporters. We observed a bacterial dose and time-dependent increase in *tnf* promoter-driven mCherry expression with the live J2315 strain, but only a very slight TNF-α reporter increase with killed bacteria (Fig. 4c). We also observed rapid p65/relA translocation to the nucleus in response to *B. cenocepacia* infection, especially at high bacterial MOI (Fig. 4d). Interestingly, while formalin killed bacteria induced very little TNF-α promoter activation, NF-κB activation was comparable to that observed with live bacteria (Fig. 4d), suggesting that the initial NF-κB translocation is not in itself sufficient to drive the high level TNF-α promoter activation observed at later time points (Fig. 4c).

### Use of constitutively expressed reporter genes for siRNA delivery optimization

**Figure 5:**
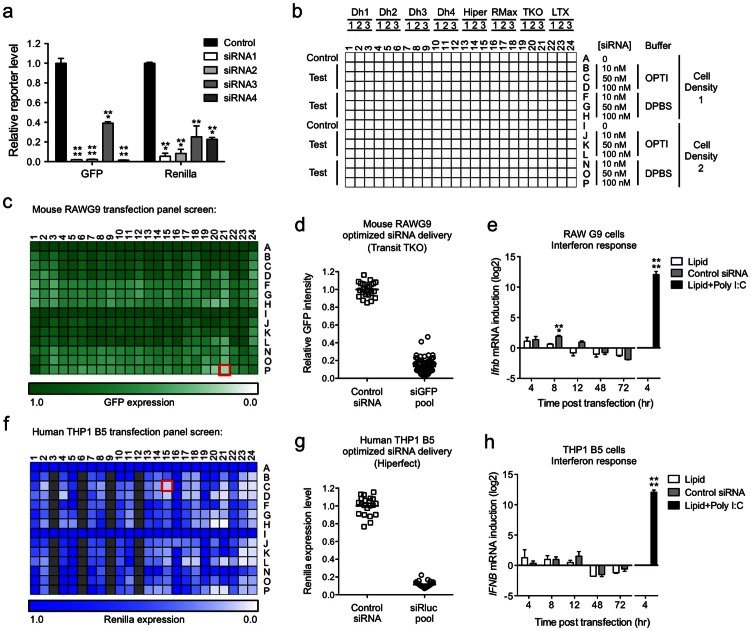
Optimization of siRNA delivery protocols for the RAW G9 and THP1 B5 macrophage reporter cell lines that avoid non-specific immunostimulatory responses. (a) Identification of optimal siRNAs targeting the GFP and renilla genes. Control or GFP/renilla siRNAs were co-transfected into HEK293 cells with GFP or renilla expression plasmids. Relative GFP or renilla reporter gene expression levels were measured at 48 hr post-transfection. GFP siRNAs 1, 2 and 4 and renilla siRNAs 1–4 were pooled for use in subsequent siRNA delivery optimization experiments in RAW G9 and THP1 B5 cells respectively (b) Experimental test matrix in 384-well format for siRNA delivery protocol optimization. Column variables are different volumes (see Methods) of the following transfection lipids: Dharmafect 1–4 (Dh1–4), Hiperfect (Hiper), RNAiMax (RMax), Transit TKO (TKO) and Lipofectamine LTX (LTX). Row variables are siRNA concentration (10–100 nM), siRNA/lipid dilution buffer (OptiMEM or dulbecco's PBS) and cell density (1 = 5,000 cells/well, 2 = 10,000 cells/well). (c) Effect on GFP-relA expression in RAW G9 reporter cells transfected with the GFP siRNA pool validated in panel a using the condition matrix in panel b measured at 72 hr post-transfection. Best knockdown with no effect on cell viability seen in plate position P21 (red square). (d) Optimized Transit TKO lipid delivery of GFP siRNA pool to RAW G9 cells in 384-well format leads to reproducible GFP-relA intensity reduction of >80%. (e) Measurement of *Ifnb* mRNA induction over time in RAW G9 cells transfected with either Transit TKO lipid alone or control siRNA via the optimized Transit TKO delivery protocol. Cells treated with lipid + 5 μg/ml poly I:C are a positive control for robust *Ifnb* induction. (f) Effect on renilla expression in THP1 B5 reporter cells transfected with the renilla siRNA pool validated in panel a using the condition matrix in panel b measured at 72 hr post-transfection. Best knockdown with no effect on cell viability seen in plate position C15 (red square). Grey squares = low cell viability. (g) Optimized Hiperfect lipid delivery of renilla siRNA to THP1 B5 cells in 384-well format leads to reproducible renilla expression reduction of >90%. (h) Measurement of *IFNB* mRNA induction in THP1 B5 cells transfected with either Hiperfect lipid alone or control siRNA via the optimized Hiperfect delivery protocol. Cells treated with lipid + 5 μg/ml poly I:C are a positive control for robust *IFNB* induction. Data are representative of two experiments (a, e, h; mean + s.d.). **P < 0.01, ***P < 0.001, ****P < 0.0001 (two-tailed t test).

Effective delivery of siRNA into hematopoietic cells remains a significant obstacle to the implementation of effective siRNA-based host pathogen RNAi screens. To establish a reproducible method that could achieve robust knockdown (KD) of target genes and reduce false negative frequency in screening, we took advantage of the fact that the reporter cell lines described above provide convenient control siRNA targets in the form of the GFP and renilla luciferase reporters they express (Fig. 1b + f). Optimizing siRNA delivery by targeting these reporters allows measurement of protein rather than mRNA KD (the latter being the most common validation method for siRNA delivery), providing a more direct assessment of the required endpoint needed for an effective screen. We first designed and tested four candidate siRNA sequences against the GFP and renilla luciferase coding sequences, and identified multiple potent siRNAs for each target (Fig. 5a). The most effective siRNAs against each target gene were pooled for testing siRNA delivery conditions in the RAW G9 and THP1 B5 reporter cells. By conducting the testing in 384-well plates we could readily examine an extensive matrix of transfection conditions (Fig. 5b), which improved the chances of identifying optimal siRNA delivery protocols for the macrophage reporter cell lines. Using this approach, we identified reproducible lipid-based transfection protocols that gave 80–95% target knockdown for both the GFP in the RAW G9 clone (Fig. 5c + d) and the renilla luciferase in the THP1 B5 reporter cell line (Fig. 5f + g). Since TLR ligand preparations can be prone to contamination with LPS, we used these optimized siRNA transfection protocols to confirm that the cell line reporter responses to the panel of TLR ligands tested in Figures 2 and 3 were not caused by trace amounts of LPS in the ligand preparations. We targeted the TLR4 gene in both the THP1 B5 and RAW G9 reporter cells with a specific siRNA pool, and found that the reporter responses were only perturbed after treatment of the cells with LPS (Supplementary Fig. 2).

Because the response to dsRNA during pathogen infection is very robust in macrophages and a strong subsequent interferon response would be problematic for the interpretation of effects resulting from gene-specific siRNA knockdown, we also looked for any interferon response to the transfected siRNA. Using the identified optimal transfection conditions, we found no significant elevation in type-I interferon production from either the RAW G9 or THP-1 B5 reporter cell lines during our assay window of 48 to 72 hr post-transfection (Fig. 5e + h).

### Validation of the mouse and human macrophage siRNA screening platform by perturbation of TLR pathway gene targets

**Figure 6:**
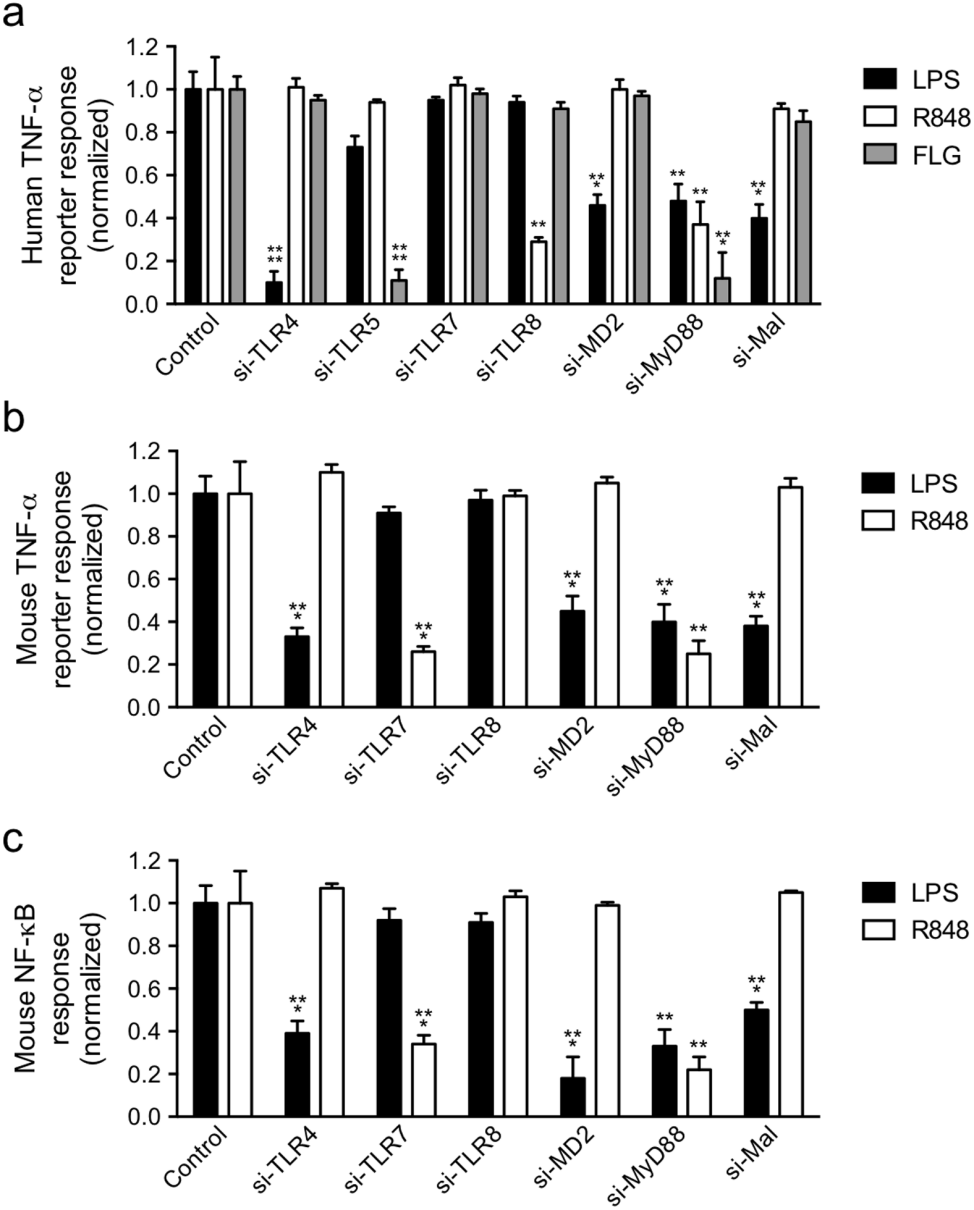
Effects of knockdown of TLR pathway genes on specific ligand responses in mouse and human macrophage reporter cell lines. (a) Human TNF-α reporter responses in THP1 B5 cells transfected with either non-targeting control or TLR pathway gene-specific siRNAs and treated for 4 hr with either 10 ng/ml LPS, 10 μg/ml R848 or 10 ng/ml FLG. (b) *tnf* promoter-driven mCherry expression at 16 hr and (c) Cytosol-to-nuclear translocation of the GFP-relA fusion at 40 min in RAWG9 cells transfected with either non-targeting control or TLR pathway gene-specific siRNAs and treated with 10 ng/ml LPS or 3 μM R848. 6 individual siRNAs per gene were used (Supplementary Table 1) and the average reporter response calculated. Data are representative of two experiments and reporter responses are normalized to the levels observed with non-targeting control siRNA (a–c; mean + s.d.). **P < 0.01, ***P < 0.001, ****P < 0.0001 (two-tailed t test).

To further assess the utility of the cell line reporters and the efficiency of the optimized siRNA delivery protocols, we targeted a range of canonical TLR pathway genes in both the human and mouse macrophage cell lines with six different siRNA per gene (Supplementary Table 1), and tested the reporter responses to TLR ligands. We observed significant reduction in the reporter responses for specific ligand/target gene combinations that were consistent with known TLR pathway architecture (Fig. 6)^1^,^5^. In the human THP1 B5 cells, we observed diminished responses to LPS with KD of TLR4, MD2, MyD88 and Mal, to R848 with KD of TLR8 and MyD88 and to flagellin with KD of TLR5 and MyD88 (Fig. 6a). In the mouse RAW G9 cells, we saw the same specific perturbation of the LPS response as seen in the human cells with KD of TLR4, MD2, MyD88 and Mal, and this was seen for both TNF-α (Fig. 6b) and NF-κB (Fig. 6c) reporters. The mouse cells also showed a perturbed R848 response with MyD88 KD, but in contrast to the human cells, TLR7 KD, but not TLR8, led to a diminished R848 response for both TNF-α and NF-κB (Fig. 6B + C). To confirm that the differential use of TLR7 and TLR8 in response to R848 observed in the mouse and human macrophage cells was not simply due to more efficient KD of the receptor for which the phenotypic effect was observed, we measured the relative efficiency of KD of both receptors in each cell type. This demonstrated that in both the RAW G9 and THP1 B5 cells, comparable KD of both TLR7 and TLR8 was achieved (Supplementary Fig. 2). This suggests that the human THP1 and mouse RAW macrophage cell lines respond to the R848 ssRNA mimic through different receptors. These results also confirm the utility of the described cell lines and siRNA delivery protocols to accurately profile gene dependencies in the TLR signaling pathway using RNAi.

## Discussion

Despite the discovery potential of RNAi technology, cells of the innate immune system pose significant challenges for RNAi application in terms of identifying conditions that achieve robust target gene knockdown while avoiding a non-specific immune response to the introduced dsRNA^7^,^12^,^13^. These challenges have led to the adoption of surrogate non-immune model cells, often with over-expressed pathogen receptors or pathway components, for host-pathogen RNAi screens. The lack of concordance in gene hits for several genome-wide screens for host factors involved in HIV^26^,^27^,^28^ and influenza^29^,^30^,^31^,^32^ may be attributed to the use of different engineered, non-hematopoietic cell lines as assay vehicles in such screens. One also cannot be certain that the hits in these screens reflect aspects of signaling pathways relevant to innate immune cells. For example, we have found that there are important mechanisms of signaling feedback control in macrophages challenged with different doses of bacterial LPS that are not evident in fibroblasts^19^.

We describe here the development of macrophage cell lines with stably integrated expression of multiple reporter genes responsive to pathogenic stimuli. These include a mouse reporter line derived from RAW264.7 cells that expresses high content imaging readouts for both NF-κB cytosol to nuclear translocation and a TNF-α transcriptional reporter, and a human line derived from THP-1 cells that combines human TNF-α promoter driven firefly luciferase with a constitutively expressed renilla luciferase that provides a normalization control for changes in cell number or transcription flux. The latter design is helpful in endpoint screening assays to filter out hits that affect cell viability or general transcriptional processes. The described cell lines are responsive to almost all TLR ligands tested, in addition to intact bacteria, proving valuable model cell systems for studies of PRR pathway dependencies in both mouse and human macrophage responses to pathogens. Moreover, the dual high content readout in the mouse RAW G9 cells also provides opportunities for live cell imaging and single cell analysis that we have previously shown can identify aspects of feedback control in NF-κB transcriptional responses and LPS dose encoding that would not have been possible from bulk cell analyses^19^.

To address the problematic nature of macrophages with respect to siRNA delivery, our use of the stably expressed reporter genes (introduced during generation of our reporter assays) as siRNA targets allowed us to test several hundred transfection conditions in a single 384-well assay plate. This showed that even notoriously difficult to transfect cells were conducive to efficient siRNA delivery if enough conditions were tested. It should be noted however that the conditions we identified in 384-well format do not necessarily scale directly into other plate formats, and we have found that it is necessary to test subtle variations of the optimized 384 well condition to achieve similar results in alternative well sizes.

The initial discovery of siRNA-based gene perturbation in mammalian cells suggested that the host interferon response to dsRNA could be avoided as long as the dsRNA shorter than 20–25 nt^8^. However, later studies demonstrated that dsRNA responses could still occur using short oligonucleotides through PRR activation in innate immune cells that are particularly sensitive to nucleic acid stimuli^12^,^13^. There are also previous reports of mouse macrophage activation in response to certain transfection lipids and to non-targeting siRNA^33^. The screening of hundreds of transfection conditions allowed us to identify siRNA/lipid combinations that showed minimal type I interferon induction and no elevation in interferon expression during the typical assay window of 24–72 hr after siRNA transfection.

Since the cell reporter systems we describe here are based on transformed cell lines, it should be noted that these cells could have peculiarities that are not reflective of the properties of primary human or mouse macrophages. It is therefore important to validate findings made in such cell lines with studies in primary cells. However, the cell lines employed here are the most widely used *in vitro* model systems for mouse (RAW264.7) or human (PMA-differentiated THP1) macrophage-like cells, and as we demonstrate, they are responsive to a wide range of TLR ligands. The described cell system is also limited to readouts of NF-κB activation and TNF-α promoter induction, however the methods used for stable reporter expression can be readily applied to additional signaling and transcriptional readouts.

In testing our described screening platforms through siRNA knockdown of select receptors and proximal signaling components in the human and mouse TLR pathways, we observed the expected specificity for TLR4 and MD2 with LPS and TLR5 with flagellin^1^. We also observed differences in the receptor requirements for the R848 response, with TLR8 required in human cells and TLR7 in mouse. This is consistent with prior observations, and may suggest species differences in how ssRNA is sensed in the endosome of human and mouse macrophages^34^,^35^. Among proximal signaling components, we identified the expected requirement of MyD88 for all ligands tested, and the selective use of Mal/Tirap in the LPS response but not for either flagellin or R848^1^.

In summary, the human and mouse macrophage reporter cells and optimized siRNA delivery protocols provide an experimental platform with significant potential for genetic screening in an innate immune cell type that is highly relevant for host pathogen studies. Furthermore, the development of cell systems in both human and mouse macrophage cell lines will permit a comparison of similarities and differences in how innate cells from the two species respond to pathogenic stimuli.

## Methods

### Generation of mouse and human macrophage reporter cell lines

Construction of the lentiviral plasmid expressing the dual GFP-RelA and *tnf* promoter mCherry-PEST reporters (Fig. 1b–e) and the infection of RAW264.7 cells with lentivirus to generate the RAW G9 cell clone has been previously described^19^. To construct the human *tnf* promoter dual luciferase reporter (Fig. 1f), the sequence expressing the renilla luciferase hRlucP gene was excised from the pGL4.71 plasmid (Promega) and used to create a lentiviral plasmid with the *ubc* promoter driving expression of hRluc2P-IRES-Neo as described previously^36^. The sequence from −1110 to −50 of the human *tnf* promoter was cloned by PCR from THP1 cells and ligated upstream of the firefly luciferase luc2P gene in the pGL4.11 plasmid (Promega). A fragment containing the human *tnf* promoter–luc2P–SV40 polyA sequence was excised from this plasmid and inserted upstream of the *ubc* promoter in the UbcP-hRluc2P-IRES-Neo plasmid described above. Recombinants were screened for the reverse orientation of the human *tnf* promoter–luc2P–SV40 polyA gene cassette 1 relative to the *ubc* promoter-hRlucP-IRES-Neo gene cassette 2 (Fig. 1a). Lentiviral particles were generated and used to infect the human THP1 monocytic leukemia cell (ATCC, TIB-20) cells as previously described. The infected cells were maintained in the presence of G418 selection followed by dilution-cloning to isolate individual cell clones. Single cell clones were isolated, expanded and screened for robust firefly luciferase response to a TLR ligand panel (Fig. 2c). A single clone (B5) was chosen for all subsequent experiments.

### High content imaging of RAW G9 mouse macrophage cells

The GFP-p65 and TNF-α promoter-mCherry reporters were imaged in the RAW G9 reporter cell clone using a BD Pathway 855 bioimager (BD biosciences). BD AttoVision software was used to automatically identify and quantify DAPI-stained cell nuclei, GFP-p65 and mCherry fluorescence. GFP located within the area of the nuclear staining (eroded by 2 pixels) was defined as nuclear NF-κB, while GFP within a 2-pixel-wide ring around the nuclear staining was defined as cytosolic NF-κB. For determination of NF-κB translocation, the ratio of nuclear to cytoplasmic GFP-p65 intensity was calculated using BD Image Data Explorer software. For mCherry expression, nuclear mCherry was quantified using the same method as for NF-κB. Background was subtracted and average intensity was used as a measure of TNF-α promoter activity. Live cell imaging of RAW G9 cells expressing GFP-p65 and *tnf* promoter driven mCherry for Supplementary Videos 1–3 was carried out as previously described^19^.

### Dual luciferase assay of THP1 B5 human macrophage cells

The human THP1 B5 cell clone was differentiated into a macrophage-like state in RPMI 1640 medium containing varying concentrations of phorbol-12-myristate-13-acetate (PMA, from Sigma, P1585) for 72 h during protocol development and with 5 ng/ml of PMA for all remaining experiments. The differentiated cells were treated with TLR ligands for activation or growth media as control. After stimulation, the cells were washed once in PBS, and lysed in passive lysis buffer (Promega). Firefly and renilla luciferase activity in the cell lysates was determined using the Dual-Luciferase Reporter Assay System (Promega, E1960), following the manufacturers protocol, and the ratio of firefly luminescence to renilla luminescence was used to reflect the cell response to stimulation.

### Cell culture and TLR ligand stimulation

RAW G9 cells were maintained in DMEM, 10% FBS, 20 mM Hepes, and 2 mM glutamine. THP1 B5 cells were maintained in RPMI1640, 10% FBS, and 2 mM glutamine containing 500 ug/ml G418. TLR ligand sources: LPS was from Alexis Biochemicals, Salmonella minnesota R595 TLRgrade, ALX-581-008-L002; Pam3CSK4 (P3C) was from EMC Microcollections, cat# L2000; PGN was from Sigma, peptidoglycan from Staphylococcus aureus, Cat.# 77140; R848 was from InvivoGen, tlrl-r848; Pam2CSK4 (P2C) was from EMC Microcollections, cat# L2020; Flagellin was from Invivogen, FLA-ST ultrapure, tlrl-epstfla; Lipid A was from Avanti Polar Lipids, 699500P; CpG was from IDT; poly I:C was from Invivogen, tlrl-picw.

### Gram-negative bacterial infection

Infection of both human THP1 B5 and mouse RAW G9 cells with *B. cenocepacia* was conducted using the infection method previously described for primary macrophages and THP-1 cells^25^.

### Optimization of siRNA delivery to RAW G9 and THP1 B5 cells

The following sequences in the EGFP and renilla luciferase coding regions were targeted with custom synthesized siRNA (Dharmacon); EGFP siRNA1 GCCACAACGTCTATATCAT, siRNA2 GCGCCGAGGTGAAGTTCGA, siRNA3 GAGCTGGACGGCGACGTAA and siRNA4 GCATCGAGCTGAAGGGCAT and renilla siRNA1 CAAGCAAGATCATGCGGAA, siRNA2 CCGAGTTCGTGAAGGTGAA, siRNA3 CAGATGAAATGGGTAAGTA and siRNA4 GGCCTTTCACTACTCCTAC. The potency of the individual siRNA sequences was assessed by co-expression in HEK293 cells with plasmids expressing GFP (pEGFP-N1 (Clontech)) or renilla (pGL4.74 (Promega)) and measuring the effect on GFP/renilla expression compared to cells transfected with a non-targeting control siRNA (NTC5; Dharmacon). Validated siRNA pools against GFP and renilla were generated by combining EGFP siRNAs 1, 2 and 4 and renilla siRNAs 1, 2, 3 and 4. The following transfection lipids and volumes (per well of 384 well plate) were tested in the optimization of siRNA delivery to RAW G9 and THP1 B5 reporter cells; Dharmafect 1 to 4/0.03–0.14 μl (Dharmacon), Hiperfect/0.125–0.25 μl (Qiagen), RNAiMax/0.025–0.075 μl and Lipofectamine LTX/0.04–0.15 μl (Life Technologies) and TransitTKO/0.04–0.2 μl (Mirus Biology LLC). Opti-MEM I and Dulbecco's phosphate buffered saline (DPBS) buffers were purchased from Life Technologies. Across the matrix of transfection conditions detailed in Fig. 5b, knockdown efficacy of GFP expression in RAW G9 cells was measured by average cytoplasmic GFP intensity quantified by high content imaging, and knockdown efficacy of renilla luciferase expression in THP1 B5 cells was measured using the Dual-Luciferase Reporter Assay System (Promega, E1960). The following optimized protocols were established for subsequent siRNA screening assays in 384-well plates: RAWG9 cells; 2 μl of 2.5 μM siRNA (final transfection concentration of 100 nM) stock was added per well. A pre-mix of 0.2 μl Transit TKO and 7.8 μl DPBS per well was prepared and added to the siRNA. Plates were shaken for 1 min and then incubated at room temperature for 20 min. 5,000 RAW G9 cells in 40 μl media were added per well and incubated at 37°C in a humidified atmosphere with 5% CO_2_ for 48 hr. Medium was replaced prior to ligand stimulation and high content assay measurement. THP1 B5 cells; 2 μl of 1.25 μM siRNA (final transfection concentration of 50 nM) stock was added per well. A pre-mix of 0.2 μl Hiperfect and 7.8 μl Opti-MEM I was prepared and incubated at room temperature for 5 min. The lipid pre-mix was added to siRNA in the well and incubated a further 20 min. To each well, 5,000 THP1 B5 cells in 40 μl cRPMI medium containing PMA (5 ng/ml final concentration) were added and incubated at 37°C in a humidified atmosphere with 5% CO_2_ for 68 hr prior to ligand stimulation and dual luciferase assay.

### Quantitative PCR (qPCR)

Total RNA was extracted with the RNeasy Micro Kit (Qiagen, 74004) and RNeasy Mini Kit (Qiagen, 74106). cDNA was synthesized from extracted RNA with iScript cDNA Synthesis Kit (Bio-rad, 170-8891). Quantitative RT-PCR was performed with Thermo Scientific Solaris qPCR Rox Master Mix (AB-4351/C) or with Power SYBR green PCR Master Mix (Applied biosystems, Cat. No. 43091655) with a Mastercycler realplex4 real-time PCR detection system (Eppendorf), according to the manufacturers' protocols. The abundances of the mRNAs of interest in each sample were normalized to that of β-actin or Hprt mRNA, and fold changes in target mRNAs relative to their basal abundances were calculated by the 2^−^ΔΔC_t_ method. The primers and probes for human and mouse TLR7 and TLR8 were purchased from GE Healthcare, Dharmacon (Solaris Mouse or Human qPCR Gene Expression Assay). The primers for human and mouse IFNβ, β-actin and Hprt were purchased from Qiagen (QuantiTech Primer Assays).

### Targeting of TLR pathway components with siRNA

TLR pathway genes were targeted with 6 unique siRNA sequences (3 each from Ambion and Qiagen) per gene (Supplementary Table S1) and individual siRNAs were arrayed in 384-well plates. Negative control siRNAs were from Dharmacon (Non-targeting controls; NTC#2, 3 or 5). LPS contamination of the TLR ligand panel was tested using siGenome SMARTpool siRNAs against human and mouse TLR4 (GE Healthcare, Dharmacon). After siRNA transfection following the optimized protocols above, THP1 B5 cells were stimulated with TLR ligands for 4 hr and subjected to dual luciferase assay. Replicate siRNA plates were run for RAW G9 cells to permit TLR ligand treatment of one plate set for 40 min (NF-κB readout) and the second plate set for 16 hr (TNF-α reporter readout). RAW G9 cells were fixed in 4% paraformaldehyde (Polyscience, Inc) and nuclei were stained with 600 nM DAPI (Invitrogen) prior to high content imaging. The reporter responses were measured for cells transfected with each of the 6 individual siRNAs/gene, and the average values were calculated.

### Microarray analysis

Total RNA was isolated from RAW G9, parental RAW264.7, THP1 B5 and parental THP1 cells with an RNeasy Mini Kit (Qiagen). Duplicate biological were run for each cell line. Amplification and labeling of complementary RNA (cRNA) was performed with the Illumina TotalPrep RNA Amplification Kit (Invitrogen), and the cRNAs were hybridized to Illumina microarrays according to the manufacturer's protocol. A detection p value threshold of 0.1 was used to predict presence or absence of TLR family genes.

## Supplementary Material

Supplementary Information Supplementary Information

Supplementary Information Supplementary Video 1

Supplementary Information Supplementary Video 2

Supplementary Information Supplementary Video 3

Supplementary Information Supplementary Table 1
