# Supplementary material for: Development of a cell system for siRNA screening of pathogen responses in human and mouse macrophages
Source: Sci Rep. 2015 Apr 1;5:9559. doi: 10.1038/srep09559 (PMC4894436; doi:10.1038/srep09559)
Supplement: Supplementary Information [file srep09559-s1.pdf]

SUPPLEMENTARY INFORMATION

Development of a cell system for siRNA screening of pathogen responses in human and mouse macrophages

Ning Li[1†], Jing Sun[1†], Zachary L. Benet[1], Ze Wang[2], Souhaila Al-Khodor[1], Sinu P. John[1], Bin Lin[1], Myong-Hee Sung[3] and Iain D.C. Fraser[1*]

[1] Signaling Systems Unit

[2] Lymphocyte Biology Section

Laboratory of Systems Biology, National Institute of Allergy and Infectious Diseases, National Institutes of Health, Bethesda, MD 20892, USA.

[3]Laboratory of Receptor Biology and Gene Expression, National Cancer Institute, National Institutes of Health, Bethesda, MD 20892, USA.

† These authors contributed equally.

* To whom correspondence should be addressed.

E-mail: fraseri@niaid.nih.gov; Tel: 301-443-5998; Fax: 301-480-5725.

a

RAW G9 clone

ARRAY PROBE_ID	EntrezID	GeneSymbol	DetectionPval	Expression call
ILMN_1236908	21897	Tlr1	0	Present
ILMN_2733733	24088	Tlr2	0	Present
ILMN_2697002	142980	Tlr3	0.4305556	Absent
ILMN_2684234	21898	Tlr4	0	Present
ILMN_2440602	53791	Tlr5	0.7382479	Absent
ILMN_1254605	21899	Tlr6	0.001068376	Present
ILMN_1245354	170743	Tlr7	0	Present
ILMN_2460572	170744	Tlr8	0.0534188	Present
ILMN_2849322	81897	Tlr9	0.09508547	Present

b

Parental RAW264.7

ARRAY PROBE_ID	EntrezID	GeneSymbol	DetectionPval	Expression call
ILMN_1236908	21897	Tlr1	0	Present
ILMN_2733733	24088	Tlr2	0	Present
ILMN_2697002	142980	Tlr3	0.6132479	Absent
ILMN_2684234	21898	Tlr4	0	Present
ILMN_2440602	53791	Tlr5	0.7596154	Absent
ILMN_1254605	21899	Tlr6	0	Present
ILMN_1245354	170743	Tlr7	0	Present
ILMN_2460572	170744	Tlr8	0.0534188	Present
ILMN_2849322	81897	Tlr9	0.09935898	Present

c

THP1 B5 clone

ARRAY PROBE_ID	EntrezID	GeneSymbol	DetectionPval	Expression call
ILMN_1731048	7096	TLR1	0.001298701	Present
ILMN_1772387	7097	TLR2	0.002597403	Present
ILMN_2155708	7098	TLR3	0.8051948	Absent
ILMN_1706217	7099	TLR4	0	Present
ILMN_1722981	7100	TLR5	0.005194805	Present
ILMN_1749287	10333	TLR6	0.006493506	Present
ILMN_1677827	51284	TLR7	0.002597403	Present
ILMN_1657892	51311	TLR8	0.001298701	Present
ILMN_1679798	54106	TLR9	0.1896104	Absent

d

Parental THP1

ARRAY PROBE_ID	EntrezID	GeneSymbol	DetectionPval	Expression call
ILMN_1731048	7096	TLR1	0	Present
ILMN_1772387	7097	TLR2	0.075974015	Present
ILMN_2155708	7098	TLR3	0.21688312	Absent
ILMN_1706217	7099	TLR4	0	Present
ILMN_1722981	7100	TLR5	0.07532466	Present
ILMN_1749287	10333	TLR6	0.013636365	Present
ILMN_1677827	51284	TLR7	0	Present
ILMN_1657892	51311	TLR8	0	Present
ILMN_1679798	54106	TLR9	0.66753245	Absent

Supplementary Figure 1

Microarray expression data showing mRNA detection calls for TLR receptors in RAW264.7 and THP1 cell lines. A detection p value threshold of 0.1 was used to predict presence or absence of TLR family genes in (**a**) RAW G9 reporter cells, (**b**) parental RAW264.7 cells, (**c**) THP1 B5 reporter cells and (**d**) parental THP1 cells.

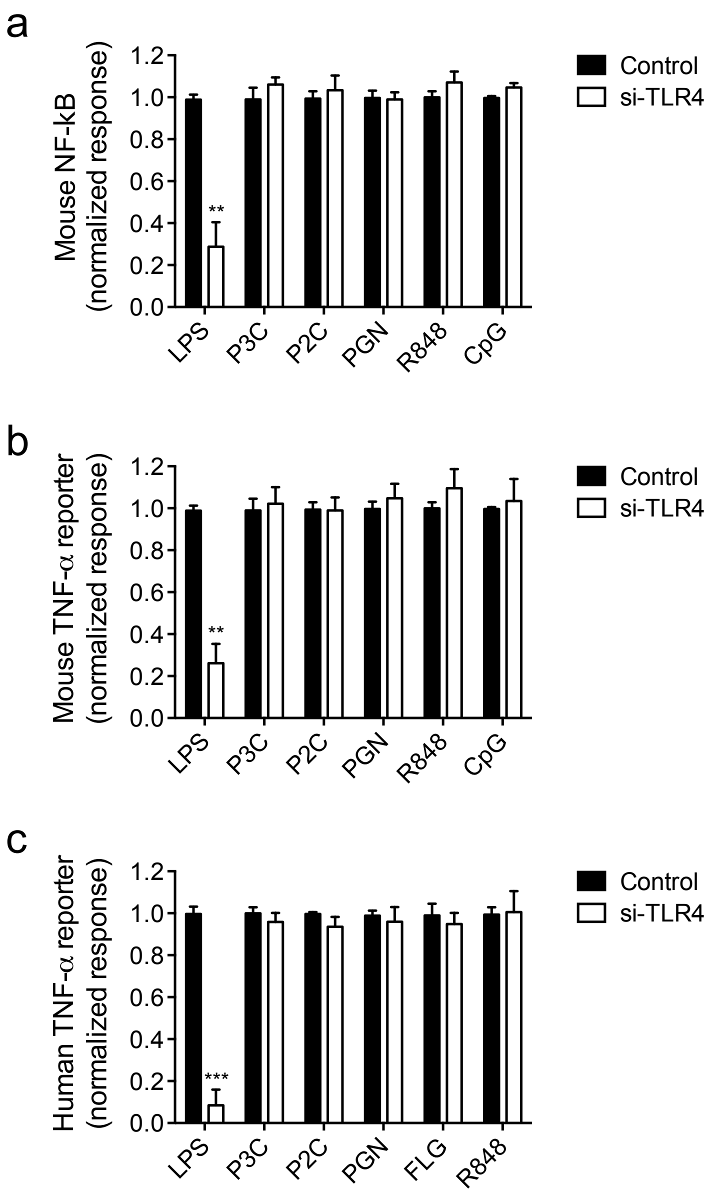

Supplementary Figure 2

Effect of knocking down TLR4 expression on mouse and human macrophage reporter responses to a panel of TLR ligands. (**a**) Cytosol-to-nuclear translocation of the GFP-relA fusion at 40 min and (**b**) *Tnf* promoter-driven mCherry expression at 16 hr in RAW G9 cells transfected with either non-targeting control or *Tlr4* gene-specific siRNA and treated with 10 ng/ml LPS, 250 nM P3C, 125 nM P2C, 100 µg/ml PGN, 3 µM R848 or 100 nM CpG. (**c**) Human TNF-α reporter responses in THP1 B5 cells transfected with either non-targeting control or *TLR4* gene-specific siRNA and treated for 4 hr with 10 ng/ml LPS, 100 nM P3C, 10 nM P2C, 10 µg/ml PGN, 10 ng/ml FLG or 10 µg/ml R848. Data are representative of two experiments (**a-c**; mean + s.d.). $^{**}P < 0.01$, $^{***}P < 0.001$ (two-tailed t test).

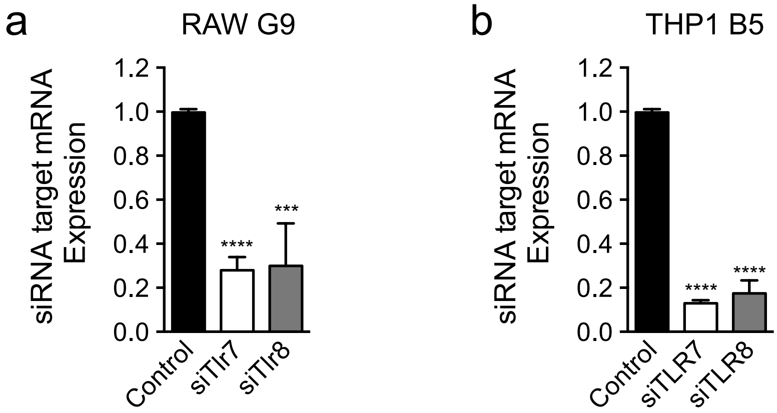

Supplementary Figure 3

Knockdown of TLR7 and TLR8 genes in mouse and human macrophage reporter cells. (**a**) RAW G9 cells were transfected with either non-targeting control, *Tlr7* or *Tlr8* gene-specific siRNA and mRNA levels were measured by qPCR. (**c**) THP1 B5 cells were transfected with either non-targeting control, *TLR7* or *TLR8* gene-specific siRNA and mRNA levels were measured by qPCR. . Data are representative of two experiments (**a, b**; mean + s.d.). ***P < 0.001, ****P < 0.0001 (two-tailed t test).

Supplementary Table 1

Details of siRNAs used to target the human and mouse TLR pathway genes. File includes gene symbols and gene ID with corresponding siRNA vendor ID and sequence information.

Supplementary Video 1

Translocation kinetics of GFP-p65/RelA in RAW G9 cells stimulated with 10 ng/ml LPS. LPS was added after acquisition of the first image and time-lapse images of a 107 μm x 107 μm field were collected every 8 min. Movie playback speed is 8 frames/sec.

Supplementary Video 2

Kinetics of *tnf* promoter-driven mCherry expression in RAW G9 cells stimulated with 10 ng/ml LPS. Movie shows red fluorescent channel of the same field of cells shown in Supplementary Video 1.

Supplementary Video 3

Kinetics of both GFP-p65/RelA translocation and *tnf* promoter-driven mCherry expression in RAW G9 cells stimulated with 10 ng/ml LPS. Movie shows combined green and red fluorescent channels of the same field of cells shown in Supplementary Video 1.
